# Addressing the Challenge of Potentially Hazardous Elements in the Reduction of Hypertension, Diabetes and Chronic Kidney Disease in the Caribbean

**DOI:** 10.5696/2156-9614-11.30.210613

**Published:** 2021-06-17

**Authors:** Adwalia Fevrier-Paul, Adedamola K. Soyibo, Nimal De Silva, Sylvia Mitchell, Chukwuemeka Nwokocha, Mitko Voutchkov

**Affiliations:** 1 Department of Physics, Faculty of Science and Technology, University of the West Indies, Mona, Jamaica; 2 Department of Medicine, University Hospital of the West Indies, Kingston, Jamaica; 3 Earth and Environmental Sciences, University of Ottawa, Ottawa, ON, Canada; 4 The Biotechnology Centre, Faculty of Science and Technology, University of the West Indies, Mona , Jamaica; 5 Department of Basic Medical Sciences, The University of the West Indies, Mona, Jamaica

**Keywords:** potentially hazardous elements, chronic kidney disease, non-communicable diseases in the Caribbean

## Abstract

**Background.:**

Environmental surveys have characterized trace elements such as lead (Pb), cadmium (Cd) and arsenic (As) as potential risk factors for non-communicable diseases. There have been few studies conducted in the Caribbean region to explore, define or clarify such findings locally. Furthermore, local pollution control efforts are often juxtaposed against more seemingly immediate economic concerns in poor communities.

**Objectives.:**

The present commentary is a call to action for the evaluation of potentially hazardous elements as potential risk indicators and/or factors of common noncommunicable diseases in the Caribbean.

**Discussion.:**

Findings from Jamaican studies have identified exposure to potentially hazardous elements (PHE) via water, food, and other anthropogenic activities to the detriment of the resident population. Several attempts have been made to abate toxic metal exposure in children with relative success. However, high levels of PHE have been noted in vulnerable populations such as patients with hypertension, diabetes mellitus and chronic kidney disease. Currently, there is low priority towards infrastructure building within the Caribbean region that would promote and sustain long term monitoring and better inform environmental polices impacting chronic diseases.

**Conclusions.:**

Further investigations are needed to clarify the role that PHE play in increasing the risk or progression of non-communicable diseases, especially in vulnerable groups.

**Competing Interests.:**

The authors declare no competing financial interests.

## Introduction

Trends in non-communicable diseases (NCD) such as chronic kidney disease (CKD), hypertension and diabetes mellitus in the Caribbean mirror the global NCD pandemic which predicts an exponential increase in incidence and prevalence over the next decade.^1^,^2^ These NCD, as well as others, are responsible for almost 70% of global deaths.^3^ At the 13th Annual Conference on Nephrology and Hypertension held in Kingston, Jamaica on January 2021, it was reported that there were currently more than 2700 CKD patients in Jamaica.^2^,^4^ Since the first lifestyle survey in 2000/2001 carried out in Jamaica, the prevalence of hypertension and diabetes has risen by 11% and over 8%, respectively.^5^,^6^ Factors such as poor dietary practices, sedentary lifestyle, tobacco smoking and excessive alcohol use have been the focus of patient management and education, and the common target for policymakers and clinicians. However, environmental contamination must be seriously investigated for its contribution to NCD.

## Discussion

The health hazards of acute exposure to potentially hazardous elements (PHE)—non essential trace elements, such as cadmium (Cd), mercury (Hg), arsenic (As) and lead (Pb) have been well documented.^7^ Several studies in Jamaica have identified potential sources of Pb, including backyard smelters and the proximity of residential districts to Pb factories and contaminated river sources, playpens and re-purposed cooking materials.^8^–^11^ There has been some success in mitigating such exposures, especially through blood lead level (BLL) screening of the pediatric population, collaboration with caregivers and primary health care workers, government policies, and community clean-up and education efforts.^8^,^12^ Overall, Pb levels in children continue to decline.^9^

However, the ongoing campaign by residents and environmentalists to prohibit bauxite mining near the Cockpit Country Protected Area highlights the most recent concerns towards pollution of ground water sources which supply 40% of Western Jamaica.^13^ While contamination in air, water and plastics are well known, the potential effects of PHE at low and chronic levels in patients with NCD are not well studied in the wider Caribbean region. The engagement of other regional stakeholders is limited without the scientific groundwork to investigate and characterize those potential effects.

Studies carried out by the International Centre of Environmental and Nuclear Sciences have indicated higher Cd levels in soil in Jamaica than found globally.^14^ The maximum naturally occurring Cd concentrations in soil exceeds 900 mg/kg, versus the global range of 0.1–0.5 mg/kg.^14^ Elevated concentrations of PHE in locally grown food products, and the kidney and liver of grazing cattle and residents in areas of high concentration, however, did not correlate directly to health indicators such as mortality rates or life expectancy.^15^,^16^ However, a study by Lalor *et al.* found that Cd was accumulated in human kidneys.^17^

Currently, research projects in Jamaica are attempting to bridge the gap of information investigating the association of PHE levels with CKD and hypertension. One such study investigated the relationship of 15 trace elements including Hg, Pb, Cd, As, selenium (Se), vanadium (V), chromium (Cr), nickel (Ni), iron (Fe) and zinc (Zn) with chronic kidney disease patients.^18^ This study revealed increased levels of toxic elements such as Cd, Pb and As, while essential elements, such as Fe and Zn were significantly depleted in patients with end stage renal disease (ESRD). Strontium (Sr), a little-known trace element in Caribbean medicine, was also surprisingly elevated in CKD patients and has been postulated as a potential biomarker for the disease.^18^ The implications for calcium substitution in the bone for Sr have been a source of debate surrounding the protective role Sr may provide against osteoporosis and paradoxically, its own contribution to Sr-induced osteomalacia.^19^ Where this may lead in improving knowledge of CKD-mineral bone disease and how this may affect the prognosis of those affected are relevant avenues for further research. To further complicate those findings, hemodialysis, the most common therapy for ESRD in Jamaica and the Caribbean, is an imperfect solution to the removal of these PHE.^18^ During hemodialysis, essential elements significantly decrease, and potentially contribute to overall deficiency in dialysis patients, while strontium levels further increase.^18^ It must be emphasized that in ESRD patients, renal excretion markedly decreases and thus is vulnerable to PHE retention within the body.^19^–^21^ This, by extension, increases exposure to nephrotoxic elements.

**Abbreviations**
*CKD*: Chronic kidney disease
*NCD*: Non-communicable disease
*PHE*: Potentially hazardous elements

Another study followed Cd measurements in the blood from study participants from every parish in Jamaica and their correlation with elevated blood pressure.^1^ The study investigated the association between blood Cd levels with blood pressure levels and the prevalence of hypertension in a representative sample of Jamaican adults living in parishes across levels of Cd in local soil. Data were collected through structured questionnaires, blood pressure readings and analyzed using spectrometric techniques. Some association between increased blood Cd levels and elevated blood pressure was observed using logistic regression analysis for Kingston/St. Andrew and St. Catherine parishes of Jamaica. This gives further credence to the involvement of environmental factors amongst the complex interactions leading to the prevalence of hypertension.

Internationally, via studies such as the United States National Health and Nutrition Examination Survey (NHANES),^22^,^23^ and its Korean counterpart (KNHANES),^24^ Cd has been identified as a possible risk factor to CKD in diabetic and hypertensive patients even at sub-level exposure. The NHANES also revealed associations of blood Pb-levels with hypertension in non-Hispanic blacks and Mexican-Americans despite declining Pb-levels.^25^ In another study, Cd and Pb were highlighted for their potential role in increasing the risk of cardiovascular disease.^26^ Arsenic exposure has been implicated as a risk factor for diabetes mellitus in several populations in Mexico,^27^ the United States,^28^,^29^ Bangladesh^30^ and Taiwan.^31^,^32^

The use of study findings to influence policy is impacted by study heterogeneity. Clinical outcomes, dietary habits, patient health status and recreational habits are not always consistently reported where these are factors that determine or influence exposure rates.^24^,^33^,^34^ Comparative analyses between studies are also limited due to differences in study design. The few nationally representative surveys which are consistently reported do not usually employ a longitudinal design. Thus, cause and effect relationships via epidemiological survey could only be approximated via conjecture as causation could not be established. Furthermore, environmental studies have been predominantly carried out in non-black populations, despite black populations being the more vulnerable racial/ethnic group prone globally to NCDs.^35^,^36^

A further challenge is presented as the outcome of PHE exposure varies based on the route/duration of exposure, biochemistry or toxicological profile of the specific contaminant, metabolic pathways altered as a consequence of exposure, and dietary habits of the population being investigated.^37^–^39^ Potentially hazardous elements tend to accumulate for long periods of time in various body systems, including bone, kidneys and liver and have sub-clinical effects in the body.^38^,^40^–^42^

The kidney, for example, as the primary filter of the body, comes into direct contact with PHE.^7^ Mercury, Pb, uranium and Cd are reported to be nephrotoxic.^43^ As the body burden of the elements increase, inflammatory mediators are recruited to sites of exposure in the form of oxidising metabolites. These metabolites then induce free radical damage on surrounding kidney cells.^44^ Chronic interstitial nephritis may be induced by Pb, and apoptosis activated by Cd. Epithelial disruption in the renal tubule by As and Cd and other pernicious effects have been attributed to oxidative damage subsequent to exposure.^38^,^45^ Potentially hazardous elements are also actively reabsorbed after glomerular filtration via metal transporters.^44^,^46^ Since many heavy metals such as silica (Si), aluminium (Al) and Sr are renally excreted, diminished kidney function increases retention of those PHE, and will exacerbate further kidney damage.^43^ With progressive kidney damage, there is activation of renin-angiotensin aldosterone system (RAAS) leading to blood pressure dysregulation and cardiac dysfunction.^47^

There may be involvement of several PHE in pathways responsible for the regulation of blood pressure and vascular permeability and ultimately endothelial damage, which will affect peripheral resistance and blood pressure auto dysregulation.^7^ Animal studies suggest increased mediators of the RAAS in response to Pb, Cd, As and Al exposure.^40^,^48^–^51^ Chen *et al.* further reported a possible link by investigating urinary As levels, high risk RAAS genetic polymorphisms and CKD.^48^ Epigenetic mechanisms exploited by PHE should be explored, especially in racially diverse populations.

Oxidative damage is facilitated by both the enhanced production of reaction oxidative species and free radicals which cause DNA and protein damage, oxidation of lipid moieties and sulfhydryl groups while depleting anti-oxidative defences.^52^ Protective biomolecules such as Vitamin C, Vitamin E, beta carotene, Se, and anti-oxidative enzymes like catalase, glutathione peroxidase, and superoxide dismutase are in high demand in chronic disease.^53^ In pre-existing health conditions, the redox balance shifts to make tissue and cellular structures vulnerable to further insults.^37^ Metals such as Cd, copper (Cu) and Ni in their composite forms have been associated with fatty acid oxidative processes and carcinogenicity.^54^ In fact, lipid peroxidation has been used as a marker for metal-induced oxidative stress.^53^ Mitochondrial structures are particularly sensitive to lipid peroxidation and is a primary target in cardiac tissue.^55^,^56^ Podocyte damage in the kidney leading to proteinuria has also been the result of mitochondrial dysfunction.^55^,^56^ The link between oxidative stress and inflammation will invariably result in exponential cellular and tissue damage, and other underlying pathophysiological mechanisms which promote development and progression of NCD.^52^

Another cause for concern in NCD is the possible relationship between malnutrition and PHE. Nutrient deficiencies contribute to the accumulation of toxic elements, since competition between heavy metals and essential trace elements can exacerbate existing trace element deficiencies.^46^ For a population susceptible to deficiencies of calcium/Vitamin D, Fe, Zn and Se, uptake by non-selective divalent metal transporters in the gut will increase the absorption of PHE such as Cd, Pb, and Hg.^38^,^44^,^46^,^57^ Consequently, the severity of health effects is linked to accumulation patterns in the body, which are often attenuated by the nutritional and dietary status of individuals.^58^–^60^ Thus, poor nutrition is one of the underlying contributors to NCD development. Furthermore, dietary restriction—a necessary intervention in CKD patients, and poor appetite—a common sequelae of disease progression—increases the risk of malnutrition, a consequence common in this patient population.^61^,^62^ Dewar *et al.* (2012), for example, found that hemodialysis patients in Jamaica were susceptible to moderate malnutrition.^63^ The understanding of these interactions is crucial to the management of NCD. Potentially hazardous element absorption and retention is another critical measure in need of further investigation.

Government agencies, private enterprises, and regulatory bodies such as the International Atomic Energy Agency should continue to pursue comprehensive policies that facilitate economic growth as well as monitor and protect long term public health concerns. Some vulnerable communities are solely dependent on backyard smelting for economic means, and chronic exposure to PHE may seem an acceptable risk. Thus, the information and infrastructure to pursue the long-term benefits of seemingly costly but practical interventions must continue to inform clinical management, public health policies and regulations to effectively minimize hazardous exposures.

## Conclusions

Variability in habitat and culture predetermine exposure rates of a population to PHE, thereby precluding the superimposition of international study findings on local and regional populations in the Caribbean. Consequently, dialogue is needed in the public health sector in Caribbean countries to investigate potential environmental hazards and related social determinants of health increasing the risk of NCDs.
